# Dose-Response Relationship Between Oral Lutein Intake and Plasma Lutein Concentration: A Randomized Controlled Trial

**DOI:** 10.3389/fnut.2022.924997

**Published:** 2022-06-22

**Authors:** Ke Xiong, Yanhui Zhao, Shouna Hu, Aiguo Ma, Yan Ma

**Affiliations:** Institute of Nutrition and Health, School of Public Health, Qingdao University, Qingdao, China

**Keywords:** lutein, dose-response relation, randomized controlled trial, carotenoid, absorption

## Abstract

Lutein was shown to provide health benefits for a few diseases. The dose-response relation of oral lutein intake in humans has rarely been reported. The objective is to investigate the dose-response relation between oral lutein intake and plasma lutein concentration in humans. Forty subjects were recruited from Qingdao University, China in 2014. The subjects were randomly divided into four groups: (1–3) consuming 10, 20, or 40 mg lutein by one, two, or four capsules of lutein A, respectively; (4) consuming 20 mg lutein by two capsules of lutein B (containing 280 mg n-3 fatty acid). After a single oral dose, plasma lutein concentrations were measured at 9-time points. The raise of plasma lutein concentration by a 40 mg dose was significantly higher than by a 10 or 20 mg dose. Plasma lutein concentrations were not significantly different between taking 20 mg lutein A and 20 mg lutein B. A dose-response relation was demonstrated between oral lutein administration and plasma lutein concentration. The dose-response relation was more pronounced among men. The current work provides a scientific basis for recommending a dietary intake level of lutein. Future work should validate the results in other ethnic and age groups.

## Introduction

Lutein is a xanthophyll carotenoid that exists in human macula lutea (1). Human obtains lutein solely from their diet (2). Lutein is typically present in green leafy vegetables such as spinach, kale, and yellow carrots (3). Lutein in nutritional supplements is typically extracted from marigold flowers (*Tagetes erecta L.*) (4). Previous randomized controlled trials showed the beneficial roles of lutein in preventing age-related macular degeneration, which was a big threat to vision in the elderly (5). Other studies suggested that lutein may also help with cognitive function (6).

Lutein absorption in the human gastrointestinal tract undergoes steps including micellization with lipids, uptake by enterocytes, and transport into chylomicrons (7). Proteins involved in the uptake, transport, and secretion of lutein affect its bioavailability which is generally very low (7–9). The recommended dietary intake level of lutein has not been established (9). A population survey estimated an intake level of 0.9–5.3 mg/d in the Western population (9). The Chinese Ministry of Health recommended an intake level of less than 12 mg of lutein ester in an official notice of 2008 (10). However, the dose-response relation of lutein in humans was rarely reported.

In this work, we conducted a randomized controlled trial in a young Chinese population to investigate the dose-response relation between oral lutein intake and plasma lutein concentration. The results would provide a theoretical basis for recommending the dietary intake level of lutein.

## Materials and Methods

### Ethics and Subjects

The study was approved by the Ethics Committee of Medicine of Qingdao Center of Disease Control and Prevention and complied with the Declarations of Helsinki. All participants provided signed informed consent. The study was registered on the Chinese Clinical Trial Registry with the registration number ChiCTR-OO-14004816.

A total of forty participants were recruited from the Medical College of Qingdao University from August to November 2014. The inclusion criteria were the following: 18–60 years old; signing an informed consent form. The exclusion criteria were the following: obesity; cardiovascular disease, hypertension, diabetes, and cancer; taking nutritional supplements such as phytochemicals, vitamins, or minerals in the past month.

### Lutein

Lutein capsule A was purchased from Vitacost.com, Inc., Boca Raton, FL, United States which consisted of 10 mg of lutein powder per capsule. Lutein capsule B was purchased from Alcon, Geneva, Switzerland, which consisted of 10 mg lutein oil and 280 mg n-3 fatty acid (including 148 mg eicosapentaenoic acid and 78 mg docosahexaenoic acid). Lutein standard was purchased from ChromaDex, Los Angeles, CA, United States. Chromatography-grade methanol and acetonitrile were purchased from Sigma-Aldrich, St. Louis, MO, United States.

### Trial Process

The forty participants were divided into four groups by a stratified randomization method which ensured a comparable gender ratio among the groups. The participants in Group A10 would take 10 mg of lutein by consuming one capsule of lutein A; the participants in Group A20 would take 20 mg of lutein by two capsules of lutein A; the participants in Group A40 would take 40 mg of lutein by four capsules of lutein A; the participants in Group B20 would take 20 mg lutein by two capsules of lutein B.

The participants were asked not to consume lutein-rich foods or foods which would affect lutein absorption from a week before the trial to the end of the trial. Common lutein-rich foods include spinach, carrot, orange, and mandarin orange; typical foods which would affect lutein absorption include fried food and alcohol. The participants were also asked to record their medication during the trial. A standard form was used to survey the basic characteristics of the participants. The height and weight of the included participants were measured by one trained project member.

The participants were asked to fast after 9 pm the day before the trial. A five mL fasting blood sample was taken at 8 am on the trial day and saved into an anticoagulant tube coated with sodium heparin. The participants were asked to consume their lutein capsules based on their group number. The blood sample was taken again at 4, 12, 24, 48, 72, 120, 168, 240, and 360 h after consuming the lutein capsules. The blood samples were immediately centrifuged at 3,000 r/min for 15 min and saved in a −80°C refrigerator until analysis.

### Plasma Lutein Concentration Analysis

Plasma lutein concentration was measured by an Agilent 1200 high-performance liquid chromatography (HPLC), Fresno, CA, United States equipped with an ultraviolet-visible light detector. A total of one hundred μL human plasma was mixed with 300 μL methanol (containing 0.1% butylated hydroxytoluene), and then vortexed for 2 min. The mixture was centrifuged twice at 13,000 r/min for 5 min. The supernatant was used for HPLC analysis. C18 column (250 mm × 4.6 mm, 5 μm) was used for separation. The column temperature was 25°C; the mobile phase was methanol and acetonitrile with a ratio of 65/35 (v/v); the flow rate was 0.8 ml/min; the injection volume was 25 μL. Lutein was detected at 446 nm. This lutein measurement method was validated in our previous publication (11).

### Statistical Analysis

Statistical analysis was performed using the Stata 17 software, College Station, TX, United States. Normal data were presented as mean ± *SD*. Mean differences among the groups were tested by an ANOVA test. A pairwise comparison was conducted using the LSD method. Mean differences at different time points within the same group were assessed by a paired *t*-test. *P* < 0.05 was considered indicative of statistical significance.

## Results

### Basic Characteristics

A total of forty participants were recruited for this study, but two of them withdrew at the beginning of the trial. The remaining 38 participants completed the trial. The basic characteristics of the 38 included participants were shown in Table 1. Age, BMI, plasma AST, and ALT concentrations were comparable among Group A10, A20, and A40. The mean age of Group B20 was slightly higher and the plasma AST and ALT concentrations of Group B20 were slightly lower than those of Group A10, A20, and A40. No one took medication during the trial.

**TABLE 1:** Baseline characteristics of the included participants.

	Group A10 (*n* = 9)	Group A20 (*n* = 9)	Group A40 (*n* = 10)	Group B20 (*n* = 10)
Age (years)	24.67 ± 1.58^a^	24.67 ± 1.12^a^	25.10 ± 2.08^ab^	26.40 ± 1.96^b^
Gender (M/F)	4/5	4/5	4/6	4/6
BMI (kg/m^2^)	21.29 ± 2.68^a^	21.59 ± 1.72^a^	20.24 ± 2.49^a^	21.89 ± 2.78^a^
ALT (U/L)	21.22 ± 6.50^a^	17.78 ± 5.52^ab^	22.60 ± 7.46^a^	13.80 ± 2.53^b^
AST (U/L)	15.78 ± 3.70^ab^	15.11 ± 2.67^ab^	17.50 ± 3.63^a^	13.70 ± 4.14^b^
ALT/AST	1.33 ± 0.20^a^	1.16 ± 0.22^ab^	1.28 ± 0.21^a^	1.04 ± 0.22^b^

*^1^The participants in group A10 would take 10 mg lutein by consuming lutein capsule A; the participants in group A20 would take 20 mg lutein by consuming lutein capsule A; the participants in group A40 would take 40 mg lutein by consuming lutein capsule A; the participants in group B20 would take 20 mg lutein by consuming lutein capsule B.*

*^2^ALT: alanine aminotransferase; AST: aspartate aminotransferase.*

*^3^The values were presented as mean ± SD.*

*Different letters in the same row indicated statistical significance (p < 0.05).*

*The mean lutein concentrations in each group were compared by an ANOVA test; a pairwise comparison was conducted using the LSD method.*

### Plasma Lutein Concentration After Oral Intake of Lutein at Different Doses

**FIGURE 1:**
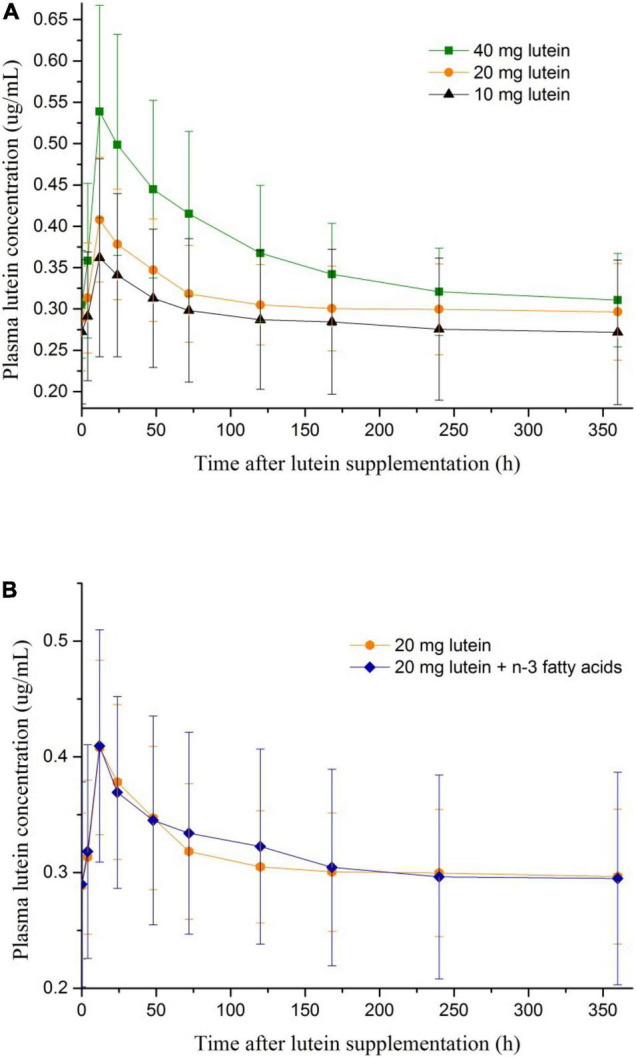
**(A)** The change of plasma lutein concentrations after lutein supplementations of different dosages. **(B)** The change of plasma lutein concentrations after lutein supplementations with and without n-3 polyunsaturated fatty acids.

The average fasting plasma lutein concentration of the 38 included participants was 0.29 ± 0.07 μg/mL. At 4–120 h after lutein intake, the plasma lutein concentration was significantly raised (*p* < 0.05) against the baseline value in each of the four groups (Table 2). The peak values of plasma lutein concentration all appeared at 12 h after lutein intake in the four groups (Table 2 and Figure 1). At 12–72 h after lutein intake, the plasma lutein concentration was significantly higher in Group A40 than those in the rest groups. The plasma lutein concentrations after lutein intake were not significantly different between the Group A20 and B20 groups.

**TABLE 2:** Comparing the plasma lutein concentrations (μg/mL) in the four groups.

Time	Group A10 (*n* = 9)	Group A20 (*n* = 9)	Group A40 (*n* = 10)	Group B20 (*n* = 10)
0 h	0.27 ± 0.09^a^	0.29 ± 0.06^a^	0.31 ± 0.07^a^	0.29 ± 0.09^a^
4 h	0.29 ± 0.08^a#^	0.31 ± 0.07^a#^	0.36 ± 0.09^a#^	0.32 ± 0.09^a#^
12 h	0.37 ± 0.12^a#^	0.41 ± 0.08^a#^	0.54 ± 0.13^b#^	0.41 ± 0.10^a#^
24 h	0.34 ± 0.10^a#^	0.38 ± 0.07^a#^	0.50 ± 0.13^b#^	0.37 ± 0.08^a#^
48 h	0.31 ± 0.08^a#^	0.35 ± 0.06^a#^	0.45 ± 0.11^b#^	0.35 ± 0.09^a#^
72 h	0.30 ± 0.09^a#^	0.32 ± 0.06^a#^	0.42 ± 0.10^b#^	0.33 ± 0.09^a#^
120 h	0.29 ± 0.08^a#^	0.31 ± 0.05^b^	0.37 ± 0.08^b#^	0.32 ± 0.08^b#^
168 h	0.28 ± 0.09^a#^	0.30 ± 0.05^a^	0.34 ± 0.06^a#^	0.30 ± 0.09^a^
240 h	0.28 ± 0.09^a^	0.30 ± 0.06^a^	0.32 ± 0.05^a^	0.30 ± 0.09^a^
360 h	0.27 ± 0.09^a^	0.30 ± 0.06^a^	0.31 ± 0.06^a^	0.30 ± 0.09^a^
AUC_(0–360 h)_	7.27 ± 4.62^a^	10.42 ± 6.99^a^	20.13 ± 9.88^b^	10.32 ± 5.82^a^

*^1^The participants in group A10 would take 10 mg lutein by consuming lutein capsule A; the participants in group A20 would take 20 mg lutein by consuming lutein capsule A; the participants in group A40 would take 40 mg lutein by consuming lutein capsule A; the participants in group B20 would take 20 mg lutein by consuming lutein capsule B.*

*^2^AUC (0–360 h): area under the curve (from time zero to 360 h), μg/mL × h; T_1/2_: half-life, h.*

*^3^The values were presented as mean ± SD.*

*Different letters in the same row indicated statistical significance (p < 0.05).*

*The mean lutein concentrations in each group were compared by an ANOVA test; a pairwise comparison was conducted using the LSD method.*

*^4^In each column, ^#^denoted statistical significance (p < 0.05) against the value at time zero, which was obtained by a paired t-test.*

**APPENDIX FIGURE 1:**
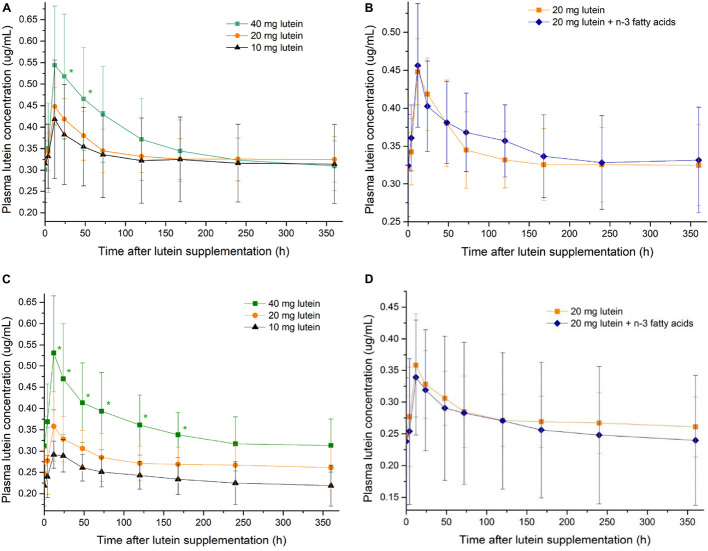
The change of plasma lutein concentrations after lutein supplementations of different dosages among women **(A)** and men **(C)**. The change of plasma lutein concentrations after lutein supplementations with and without n-3 polyunsaturated fatty acids among women **(B)** and men **(D)**. *indicates statistical significance against the 10 mg lutein group (*P* < 0.05).

Subgroup analyses stratified by gender (Appendix Figure 1) indicated that: among men, the plasma lutein concentration was significantly higher in Group A40 than those in the rest groups at 12–168 h after lutein intake; among women, the statistically significant difference was only observed at 24–48 h after lutein intake. The dose-response relations were more pronounced among participants who are men.

### Areas Under Plasma Lutein Response Curves

**FIGURE 2:**
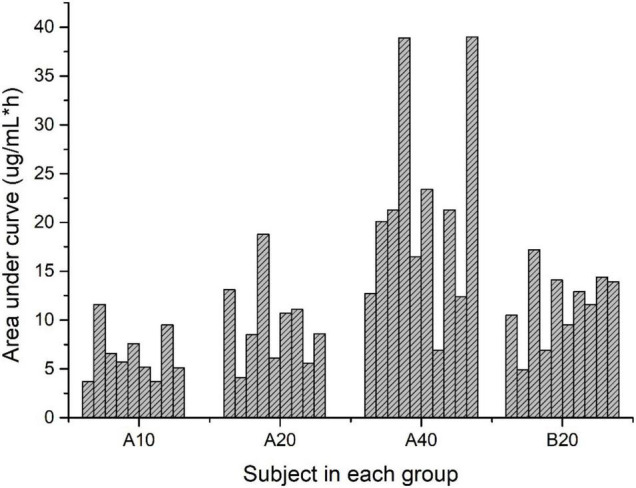
Area under the curve for lutein concentration over time (μg/mL × h) for subjects in each group.

The area under the curve (AUC) from time zero to 360 h [AUC_(0–360h)_] was significantly higher in Group A40 than those in the rest groups. AUC_(0–360h)_ was similar between the Group A20 and B20 groups. Figure 2 showed the individual variability of AUC_(0–360h)_ among the subjects in each group. A broad range of AUC_(0–360h)_ (6.9–39 μg/mL × h) was observed in Group A40.

## Discussion

To our knowledge, the current work is one of the first studies to investigate the dose-response relationship between oral lutein intake and plasma lutein concentration in humans. A single oral dose of 40 mg of lutein more significantly improved plasma lutein concentration than a single oral dose of 10 mg and 20 mg of lutein. The dose-response relation was more pronounced among men. N-3 fatty acid did not help improve lutein absorption.

The average baseline fasting plasma lutein concentration was 0.29 ± 0.07 μg/ml in the current work, which was higher than the literature values (1, 12). Green et al. reported an average baseline plasma lutein concentration of 0.11 ± 0.05 μg/ml (1), and Borel et al. reported an average baseline plasma lutein concentration of 0.17 ± 0.01 μg/ml (12). Lutein bioavailability was reported to be associated with a combination of single nucleotide polymorphisms (SNPs) (12). In literature, plasma lutein concentration values were mostly reported from the Western population, which may have different SNP expressions compared with the Chinese population as evaluated in the current work (12).

The peak values of plasma lutein concentration by three dosages of oral lutein intake all appeared at 12 h after oral administration, similar to what was reported previously (13). Dosage appeared not to affect the peak time of plasma lutein concentration after oral intake. The mean ratios of the peak concentration to the initial concentration after a single dose of 40, 20, and 10 mg lutein were 1.9, 1.4, and 1.3, respectively, in our work, which is smaller than the ratio reported by Kostic et al. by a single dose of 17 mg lutein (mean ratio of 2.9) (13). The difference may be related to a higher baseline plasma lutein concentration in our work than in the work by Kostic et al. (0.29 ± 0.07 μg/ml vs. 0.17 ± 0.06 μg/ml) (13). In addition, more pronounced dose-response relations between oral lutein intake and plasma lutein concentrations were observed among men compared with women in our work, indicating potentially different metabolism of lutein between genders.

The plasma lutein concentration returned to the baseline level 120 h After oral administration in our work, much quicker than what was reported previously (440 h after oral administration) (13). We performed a paired *t*-test to compare the plasma lutein concentration at 120 vs. 0 h after oral intake of lutein, while the previous work did not conduct such statistical analysis. As we reported previously, a continuous increase of plasma lutein concentration can be achieved by a daily oral intake of 20 mg lutein for 20 days, and the average peak plasma lutein concentration reached 1.11 μg/mL (14). Oral intake of lutein supplement is an efficient way to raise plasma lutein concentration.

The average areas under plasma lutein concentration curves after the single dose of lutein (20.13, 10.42, and 7.27 μg/mL × h for the single dose of 40, 20, and 10 mg lutein) were lower than reported by Kostic (33.86 μg/mL × h) (13). This difference and the large individual variability among the subjects, particularly in Group A40, may be associated with the different expressions of SNPs related to lutein bioavailability (7, 12).

Consistent with previous work (15, 16), the current results indicated that n-3 fatty acid did not improve lutein bioavailability. Oleic acid-rich triacylglycerol was shown to promote lutein bioavailability (17). The degree of unsaturation and the position of the carbon-carbon double bond affected the micellization of lutein during absorption, which ultimately influenced lutein bioavailability (18). Recent literature showed that mono- and di-glyceride further improved lutein bioavailability compared with oleic acid-rich safflower oil (19).

The study’s limitations should be acknowledged. First, the current work was conducted on the Chinese population. Lutein absorption was demonstrated to be associated with a few human SNPs, which would be affected by genetic background. The validity of the obtained dose-response relation should be confirmed in other ethnic populations in the future. Second, a young population was included in the investigation. Other age groups particularly the elderly should be studied in the future.

In conclusion, a dose-response relation between oral lutein intake and plasma lutein concentration was demonstrated in a young Chinese population. The dose-response relation was more pronounced among men. Oral lutein intake is an applicable method to raise plasma lutein concentration. Future studies should validate these results in other ethnic and age groups.

## Data Availability Statement

The raw data supporting the conclusions of this article will be made available by the authors, without undue reservation.

## Ethics Statement

The studies involving human participants were reviewed and approved by the Ethic Committee of Medicine of Qingdao Center of Disease Control and Prevention. The patients/participants provided their written informed consent to participate in this study.

## Author Contributions

YM and AM designed the study. YZ conducted the experiments. KX and SH analyzed the data. KX and YM wrote the manuscript. All authors read and approved the final manuscript.

## Conflict of Interest

The authors declare that the research was conducted in the absence of any commercial or financial relationships that could be construed as a potential conflict of interest.

## Publisher’s Note

All claims expressed in this article are solely those of the authors and do not necessarily represent those of their affiliated organizations, or those of the publisher, the editors and the reviewers. Any product that may be evaluated in this article, or claim that may be made by its manufacturer, is not guaranteed or endorsed by the publisher.
